# Good outcome after neoadjuvant chemotherapy and extended surgical resection for a large radiation-induced high-grade breast sarcoma

**DOI:** 10.1186/1477-7800-3-18

**Published:** 2006-07-07

**Authors:** Claudio Almeida Quadros, Alessandro Vasconcelos, Roque Andrade, Rogério Santos Ramos, Eduardo Studart, Geraldo Nascimento, André Trajano

**Affiliations:** 1 Surgical Oncologist, Oncological Society of Bahia – ONCO, Salvador, Bahia, Brazil; 2 Clinical Oncologist, Oncological Society of Bahia – ONCO, Salvador, Bahia, Brazil; 3 Plastic Surgeon, Federal University of Bahia, Salvador, Bahia, Brazil; 4 Pathologist, Silvany Studart Pathology Laboratory, Salvador, Bahia, Brazil; 5 Thoracic Surgeon, Portuguese Hospital, Salvador, Bahia, Brazil

## Abstract

This article is a case report of a high grade, radio-induced, breast malignant fibrous histiocytoma (undifferentiated high grade pleomorphic sarcoma), which developed in a 44-year old female, seven years after breast conservative surgery and radiotherapy for a T1N0M0 invasive left breast ductal carcinoma. The sarcoma presented as a fast growing tumour, 9.5 cm in the largest diameter, with skin, left breast, chest wall muscle and rib invasion.

Neoadjuvant chemotherapy was performed with epirubicin and ifosfamide. Extended radical surgery according to oncological standards and soft tissue reconstruction were carried out. Despite bad prognostic features of high grade and large invasive sarcoma, the patient is currently, after 44 months of follow up, without local recurrence, or metastases, exceeding the 12.8-month mean recurrence period and mortality rate for these tumours larger than 8.1 cm (± 1.2 cm) as described in the literature.

## Background

External radiotherapy has now become standard following breast conservative surgery. Despite this, the occurrence of radiation-induced sarcoma is still reported as a rare complication, with incidence ranging from 0.008% to 0.48% [1-4]. Radio-induced breast sarcoma has a poor prognosis with a 68% recurrence rate after resection, and mean recurrence period of 12.8 months (median = 7.5 months) [5]. Regarding survival, reported mortality rate is 67.6% at a mean of 61.4-month follow-up [5], with a five-year survival rate ranging from 27.7% to 36% [4,6,7].

Angiosarcoma, malignant fibrous histiocytoma and oesteosarcoma are the most frequent histopathogical types of radiation-induced breast sarcoma published in the English-language literature [4-6,8,9].

## Case report

**Figure 1:**
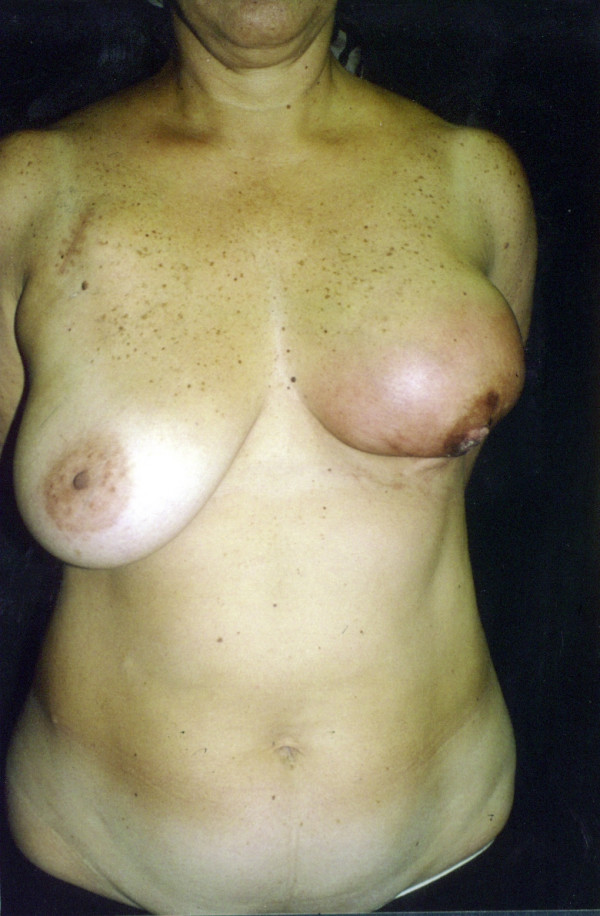
Radio-induced breast sarcoma, front view.

**Figure 2:**
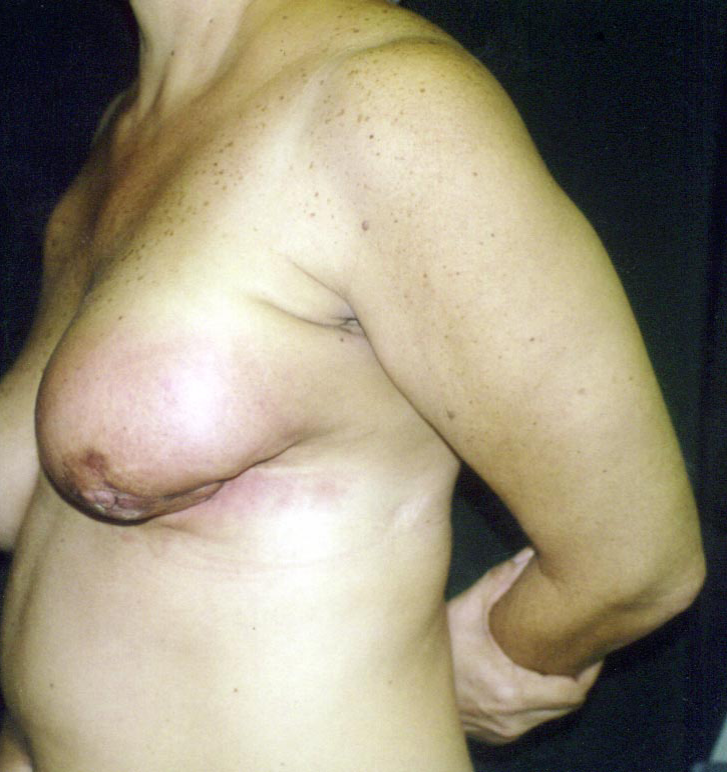
Preoperative lateral view.

**Figure 3:**
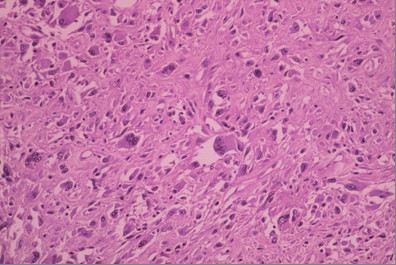
Malignant fibrous histiocytoma (hematoxylin and eosin section).

A 44-year-old woman was admitted in May 2002 at our unit with a fast growing tumour in her left breast. Clinical evaluation revealed a lesion more than 10 cm in its maximum diameter, occupying the whole breast. The tumour had grossly infiltrated the skin and was fixed to the chest wall (figure 1 and 2). Incisional biopsy revealed the tumour to have high-grade spindle cell morphology and negative epithelial immunohistochemical markers, compatible with sarcoma (figure 3).

**Figure 4:**
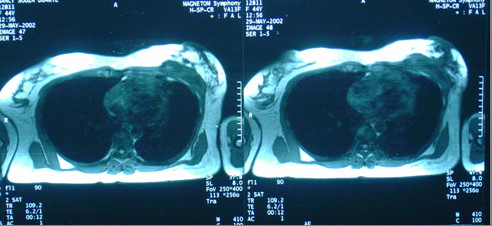
Thorax Magnetic Resonance Imaging (MRI) showing tumor invading anteriorly the skin and posteriorly ribs and pleura.

**Figure 5:**
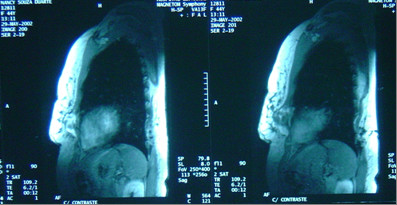
Sagittal view of thorax MRI, tumor invading breast and chest wall.

Subsequent thoracic magnetic resonance imaging (MRI) revealed an expansive lesion of left breast and chest wall origin, infiltrating the skin and invading the pectoralis major and minor muscles, and the 7^th ^– 9^th ^ribs (figures 4 and 5). Thoracic and abdominal Computed Tomography did not identify any metastases.

The patient had been treated seven years earlier for a left breast ductal carcinoma T1N0M0 invasive, and well differentiated. The treatment had consisted of a local 1.5 cm tumour excision in the left superior quadrant of the left breast with ipsilateral axillary lymph node dissection. Histological examination of the 29 lymph nodes excised was negative for malignant cells. Post-operative external beam radiotherapy was initiated 27 days after the surgery. It was applied to the remaining left breast, left axilla, supraclavicular fossa and internal mammary chain lymph nodes. The total 55 Gy radiation dose was delivered in 25 fractions, during a 5-week period, using a 3-field technique, in a Cobalt unit. The treatment ended in August 1994 with no adjuvant chemotherapy, and she had remained disease-free.

Neoadjuvant treatment for the high-grade sarcoma was initiated in June 2002, together with intravenous epirubicin, in a bolus dose of 60 mg/m^2 ^from day D1 to D2, and ifosfamide, intravenous dose of 1800 mg/m^2 ^from day D1 to D5 [9,10]. Anaemia, leucopoenia, nausea, vomiting and diarrhoea were the main adverse effects, with decreased leukocyte counts to 400 per mm^3^. Four sessions of chemotherapy were taken with 21-day intervals. At the end of the second session, clinical evidences of approximately 20% decrease in tumour diameter and resolution of the adjacent satellite lesions were observed.

**Figure 6:**
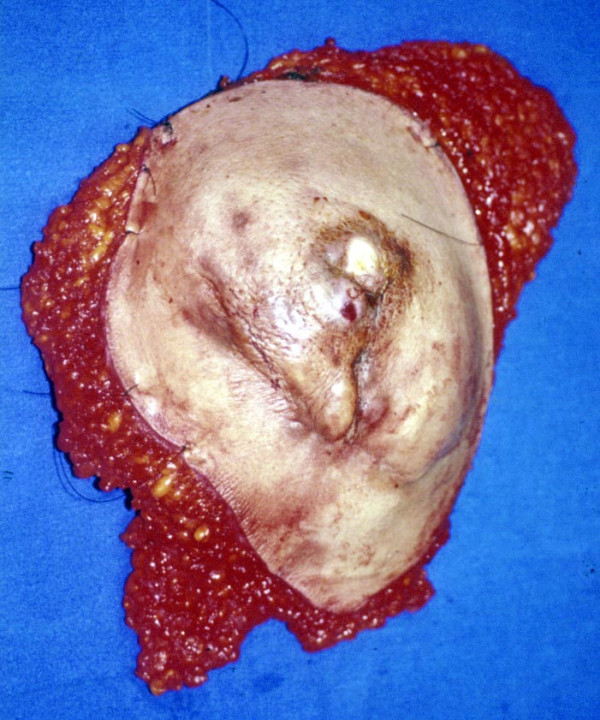
Anterior view of the surgical specimen showing skin invasion.

**Figure 7:**
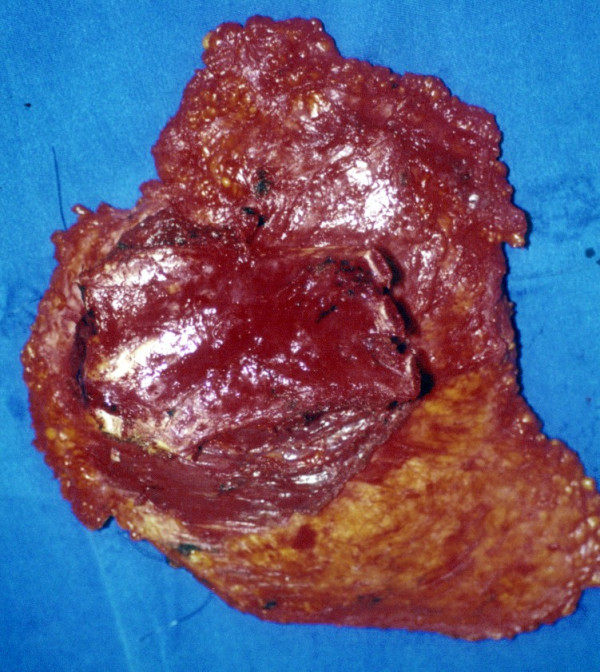
Ribs resection in the posterior view of the surgical specimen.

Surgery was performed in October 31^st^, 2002, according to oncological standards. Total radical mastectomy with chest wall en bloc resection was carried out. The oncological principle was to totally excise the tumour-infiltrated tissue with cancer cell-free margins of at least 2 cm. A large portion of the skin extending from the sternum to the left axilla and from the left clavicle to the 10^th ^left rib was resected. As the dissection was taken deep, the breast, pectoralis major and minor muscles, part of the serratus anterior muscle and the 7^th ^to 9^th ^ribs and pleura were removed en bloc (figure 6 and 7). A large soft tissue and chest wall defect required surgical reconstruction.

**Figure 8:**
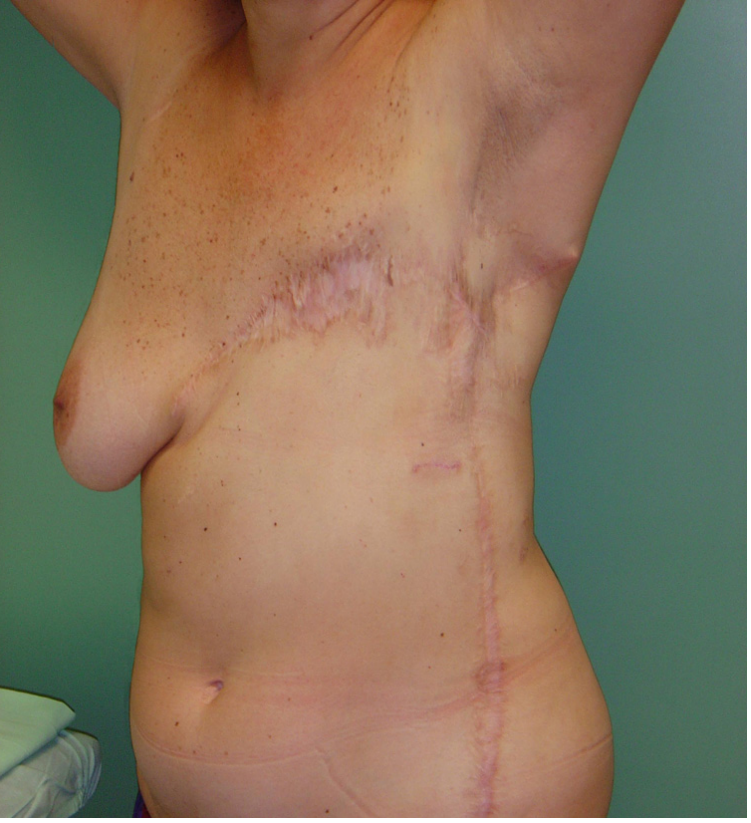
Result of radical surgical resection with oncological standards and reconstruction.

The principles of chest wall reconstruction were to provide obliteration of intra-thoracic dead space, skeletal stabilization and adequate soft tissue coverage. A 20 × 10 cm prolene mesh, covered by transposition and rotation of well vascularized regional muscle flaps, was used for skeletal stabilization. The mobilized muscles were the contralateral pectoralis major, keeping free the serratus anterior and the ipsilateral latissimus dorsi muscles. Adequate soft tissue coverage was achieved advancing and rotating an extensive thoracic-abdominal fasciocutaneous flap (figure 8). The reconstruction maintained normal respiration physiology, protected thoracic viscera and provided soft tissue coverage.

Histopathological examination of the surgical specimen confirmed a 9.5 × 9.0 × 8.5 cm high-grade breast and chest wall sarcoma invading the skin anteriorly and three ribs, pectoralis muscles and portion of the serratus muscle posteriorly. Histological evaluation revealed marked cytological and nuclear pleomorphism, often with bizarre tumour giant cells, occasionally multinuclear, combined with spindle cells and necrosis. Immunohistochemical examination showed tumour cells were reactive for vimentin. Stains were negative for cytokeratin (AE1/AE3), actin (HHF-35, IA4), calponin, myo-D1, S100 protein, CD34 and CD31. These immunohistochemical and histopathological findings allowed a diagnosis of undifferentiated high-grade pleomorphic sarcoma (pleomorphic malignant fibrous histiocytoma).

**Figure 9:**
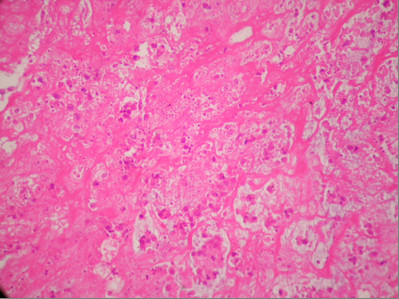
Presence of necrosis in 20% of the surgical specimen after neoadjuvant chemotherapy.

Presence of 20% of necrosis in the surgical specimen was an important finding of the histological examination, which had not been observed at incisional biopsy performed prior to neoadjuvant chemotherapy (figure 9).

The patient is currently in good health with no recurrence and breast reconstruction is being planned.

## Discussion

It is well known that ionizing radiation can induce sarcoma [1,8]. Cahan et al in 1948 defined the criteria for post-radiation bone sarcoma [12]. The requirements are: (a) evidence of an initial distinct malignant tumour different from the subsequent sarcoma, (b) development of the second malignant tumour in an irradiated field, (c) long interval between irradiation and development of sarcoma, and (d) histological confirmation of sarcoma [9]. This case report fulfils all these criteria.

Prognostic factors leading to poor outcome in breast radio-induced sarcomas are large and high-grade tumours, as described previously. In a study with 34 individuals with localized disease at the time of the surgery, tumour mean diameters of 7.9 cm ± 0.9 (n = 11) had malignant recurrences, with 12.8-month ± 10.6 (median = 7.5 months) mean recurrence period, while those with mean diameters of 3.3 cm ± 1.5 (n = 4) did not (p = 0,021) [5]. Patients who were alive without evidence of cancer had a mean tumour size of 4.2 cm ± 0.77, whereas those who died of the disease had mean tumour size 8.1 cm ± 1.2 tumours (p = 0.030) [5]. In a series of 80 radio-induced sarcoma patients, composed of 42% of breast treated patients, histological grade 2 sarcomas had a 5-year survival rate of 62%, compared to 19% survival rate in grade 3 (high grade) sarcomas (p < 0.01) [8].

The unsatisfactory results of recurrence and mortality rates of large and high grade radio-induced breast and chest wall sarcomas may have led oncologists worldwide not to consider extended surgical resection. Reasons include literature evidence of unfavourable prognosis, patient morbidity caused by the amount of removed surgical tissue. Palliative chemotherapy has been the mainstay of management in these situations. This case report indicates that patients with large and high-grade radiation-induced breast sarcomas can be offered a curative treatment. The reported patient has already survived 44 months of follow-up without evidence of malignancy, despite the presence of bad prognostic features, surpassing the recurrence range period described in the literature for patients with high-grade and large (9.5 cm) tumours [5].

## References

[B1] Huang J, Mackillop WJ (2001). Increased risk of tissue sarcomas after radiotherapy in women with breast carcinoma. Cancer.

[B2] Karlsson P, Holmberg E, Samuelsson (1998). Soft tissue sarcoma after treatment for breast cancer – a Swedish population-based study. Eur J Cancer.

[B3] Taghian A, de Vathaire F, Terrier P (1991). Long-term risk of sarcoma following radiation treatment for breast cancer. Int J Radiat Oncol Biol Phys.

[B4] Kirova YM, Vilcoq JR, Asselain B (2005). Radiation-Induced Sarcomas after Radiotherapy for Brest Carcinoma. A large-scale single-institution review. Cancer.

[B5] Blanchard DK, Reynolds C, Grant CS (2002). Radiation-induced breast sarcoma. Am J Surg.

[B6] Yap J, Chuba PJ, Thomas R (2002). Sarcoma as a second malignancy after treatment for breast cancer. Int J Radiation Oncology Biol Phys.

[B7] Brady MS, Garfein CF, Petrek JA (1994). Post-treatment sarcoma in breast cancer patients. Ann Surg Oncol.

[B8] Lagrange JL, Ramaioli A, Chateau MC (2000). Sarcoma after radiation therapy: retrospective multi-institucional study of 80 histologically confirmed cases. Radiology.

[B9] Monroe AT, Feigenberg SJ, Mendenhall NP (2003). Angiosarcoma after breast-conserving therapy. Cancer.

[B10] Meric F, Milas M, Hunt KK (2000). Impact of Neoadjuvant Chemotherapy on postoperative morbidity in soft tissue sarcomas. J Clin Oncol.

[B11] Frustaci S, Gherlinzoni F, De Paoli A (2001). Adjuvant chemotherapy for adult soft tissue sarcomas of the extremities and girdles: results of the Italian randomized cooperative trial. J Clin Oncol.

[B12] Cahan WG, Woodward HQ, Higinbotham NL (1948). Sarcoma arising in irradiated bone. Cancer.

